# CCR6 is required for ligand-induced CatSper activation in human sperm

**DOI:** 10.18632/oncotarget.20651

**Published:** 2017-09-05

**Authors:** Ruiying Diao, Tao Wang, Kin Lam Fok, Xiaofeng Li, Yechun Ruan, Mei Kuen Yu, Yimin Cheng, Ying Chen, Hao Chen, Lisha Mou, Xueyong Cai, Yan Wang, Zhiming Cai, Xuhui Zeng, Hsiao Chang Chan

**Affiliations:** ^1^ Shenzhen Key Laboratory of Genitourinary Tumor, Shenzhen Second People’s Hospital, First Affiliated Hospital of Shenzhen University, Shenzhen, China; ^2^ Institute of Life Science and School of Life Science, Nanchang University, Nanchang, China; ^3^ Epithelial Cell Biology Research Center, School of Biomedical Sciences, Faculty of Medicine, The Chinese University of Hong Kong, Hong Kong SAR, China; ^4^ Interdisciplinary Division of Biomedical Engineering, The Hong Kong Polytechnic University, Hong Kong SAR, China; ^5^ Shenzhen Research Institute, The Chinese University of Hong Kong, Shenzhen, China

**Keywords:** CCR6, CatSper, human β-defensin-1, progesterone, sperm

## Abstract

CatSper channel has been considered the principal sperm Ca^2+^ channel responsible for the cytosolic Ca^2+^ elevation required for various sperm functions necessary for fertilization [1–4]. However, the mechanism underlying the activation of CatSper channel by various physiological ligands remain incompletely understood. We have recently demonstrated the expression of C-C chemokine receptor 6 (CCR6) in sperm and Ca^2+^ influx upon binding of human β-defensin 1 (DEFB1) to CCR6, which is important for sperm motility [5]. In the present study, we have demonstrated that CCR6 receptor and CatSper channel are both required for the Ca^2+^ entry/current induced by physiological ligands DEFB1, chemokine (C-C motif) ligand 20 (CCL20) and progesterone in human sperm. CCR6 is co-localized and interacts with CatSper in human sperm. Ca^2+^ influx mediated by CCR6 and CatSper is required for essential sperm functions, including motility, hyperactivation and acrosome reaction, which are impaired in infertile sperm showing reduced levels of CCR6 and CatSper. The present finding suggests a critical role of CCR6 receptor in mediating ligand-induced, CatSper-dependent Ca^2+^ influx required for various sperm functions and thus male fertility.

## INTRODUCTION

CatSper channel is a sperm Ca^2+^ channel consisting of four pore-forming subunits CatSper1-4 and three auxiliary subunits CatSper β, γ and δ [3, 6–9]. Spermatozoa from mice with knockout of individual CatSper subunits 1-4 have decreased motility and fail to undergo hyperactivation [2, 10, 11]. As a result, these spermatozoa are unable to fertilize the egg, leading to the sterile phenotype [2, 10, 11]. In human sperm, the CatSper channel has been shown to mediate the Ca^2+^ influx induced by progesterone [12, 13], which can be released from the outer layer of the egg, the cumulus cells, and activate sperm hyperactivation and acrosome reaction [14, 15]. Recent studies further shown that orphan enzyme ABHD2 act as a steroid-selective, progesterone-dependent lipid hydrolase that depletes endocannabinoid 2-arachidonoylglycerol (2AG) to activate CatSper channel [16, 17]. Apart from progesterone, another ligands odorants directly activate CatSper through cAMP-independent pathway [18]. These results suggest the polymodal nature of the CatSper channel in sensing various ligands involved in regulating sperm functions. However, the mechanism underlying the activation of CatSper channel by various ligands remain largely unknown.

CCR6 was first identified on lymphocytes and subsequently shown to be expressed by immature dendritic cells and memory T cells for adaptive immunity [19]. Three different ligands have been shown to bind to CCR6: macrophage inflammatory protein-3alpha (MIP-3alpha; or CCL20), human β-defensins 1 and 2 (DEFB1 and DEFB2)[20]. Recent studies have shown that CCR6 is also expressed in sperm [5, 21] and we have also demonstrated that the binding of DEFB1 to CCR6 elicits a Ca^2+^ elevation in human sperm required for their motility [5]. Although it was not clear what type of Ca^2+^ channel was involved in mediating the DEFB1-induced Ca^2+^ entry, this Ca^2+^ mobilization could be abolished by specific antibody against CCR6 [5]. Since CatSper is considered the principal Ca^2+^ channel in sperm, we suspected its involvement in mediating the DEFB1-induced, CCR6-mediated Ca^2+^ mobilization. We undertook the present study to examine its importance in sperm functions and male fertility in human sperm from normal subjects and infertile patients.

## RESULTS

### DEFB1-induced Ca^2+^ mobilization requires CatSper channel

**Figure 1:**
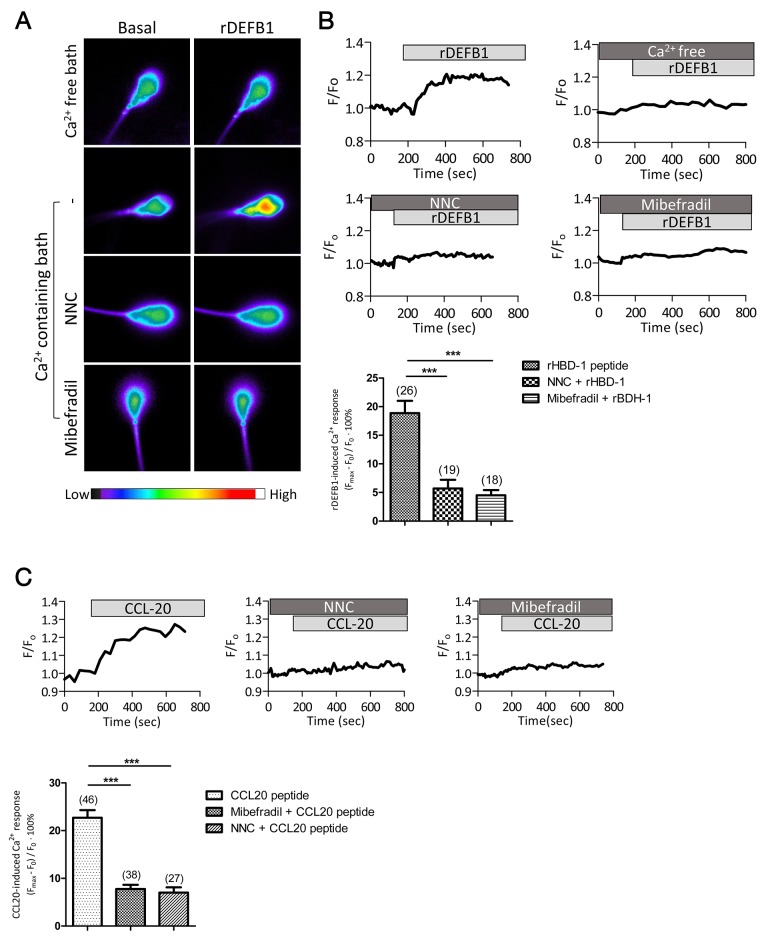
DEFB1-induced Ca^2+^ influx in human sperm depends on both CCR6 receptor and CatSper channel **(A)** Representative fluorescence images of human sperm loaded with Fluo-4, a Ca^2+^ sensitive dye, before (basal) and after the addition of rDEFB1 (800 ng/ml) into Ca^2+^-free or Ca^2+^-containing bath in the absence or presence of CatSper inhibitors, NNC55-0396 (NNC, 2 μM) or mibefradil (40 μM), with color code bar shown below. **(B)** Representative time-course change in Fluo-4 intensity (F) normalized to the initial intensity (F_0_) in the human sperm under different treatments (indicated by bars on the top). *Bottom,* statistic summary of the rDEFB1-induced Ca^2+^ responses as indicated by the difference (in % of F_0_) between F_0_ and the maximal fluorescence intensity (F_max_) achieved after the addition of rDEFB1. Data are presented as mean ± SEM. Number of measured sperm is show in each column (*** p<0.001, One-way ANOVA). **(C)** Time-course change in Fluo-4 (F) normalized to F_0_ in human sperm in response to CCL20 (50 ng/ml) in the presence or absence of NNC (2 μM) or mibefradil (40 μM). *Bottom panel,* statistic summary of CCL20-induced Ca^2+^ responses (in % F_0_). Data are presented as mean ± SEM. Number of measured sperm is show in each column (*** p<0.001, One-way ANOVA).

To test the potential involvement of CatSper channel in DEFB1-induced Ca^2+^ mobilization, we first examined the DEFB1-induced Ca^2+^ mobilization in the head region using a Ca^2+^ sensitive fluorescent probe since CatSper-mediated Ca^2+^ influx can trigger a Ca^2+^ propagation toward the sperm head [22]. Recombinant DEFB1 (rDEFB1; 800 ng/ml) elicited a sustained elevation of intracellular Ca^2+^ level ([Ca^2+^]_i_) in Ca^2+^-containing medium compared to Ca^2+^-free medium (Figure 1A-1B), suggesting that DEFB1 triggers Ca^2+^ influx. A neutralizing antibody against CCR6 receptor (20 μg/ml) [5], which targets amino acid position 151-231 that includes an extracellular motif, almost completely blocked the Ca^2+^ influx induced by rDEFB1 while control IgG did not have significant effect (Figure 1A-1B), indicating the requirement of CCR6 in mediating the Ca^2+^ response. Similarly, CatSper channel inhibitors, NNC 55-0396 or mibefradil [12, 13], used at 2 μM and 40 μM, mimicked the effect of CCR6 antibody and resulted in nearly complete blockage of the [Ca^2+^]_i_ increase induced by rDEFB1 (Figure 1A-1B). These results suggest that the DEFB1-induced Ca^2+^ influx is dependent on both CCR6 and CatSper.

Next, we tested if CCL20, another CCR6 ligand found in follicular fluid and seminal plasma [21], could similarly lead to CatSper-mediated Ca^2+^ influx. Indeed, treatment of sperm with CCL20 (50 ng/ml) also triggered Ca^2+^ influx, which could be blocked by either CCR6 antibody or CatSper inhibitors (Figure 1C). Taken together, these results suggest that physiological ligands of CCR6, either DEFB1 or CCL20, can induce CatSper-dependent Ca^2+^ influx in human sperm.

### CCR6 receptor ligands induce CatSper channel activity

**Figure 2:**
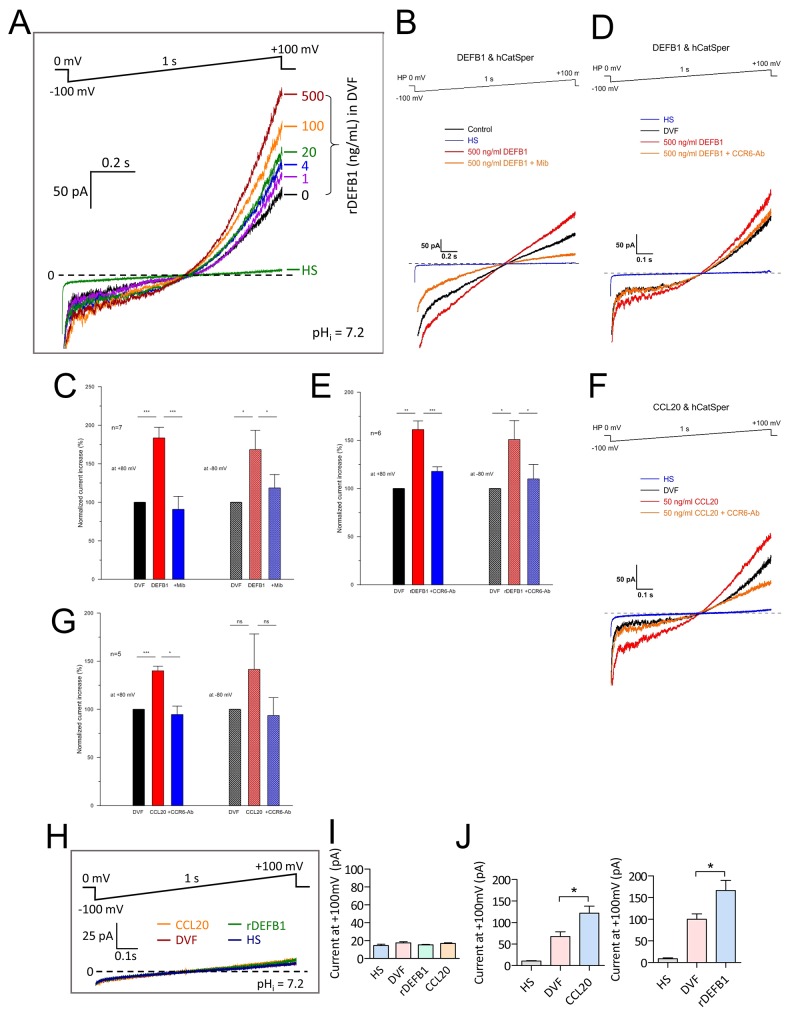
Activation of CatSper currents by CCR6 ligands in human sperm **(A-G)** Representative patch-clamp recording (A, B &D) of whole-cell currents in normal human sperm elicited from a holding voltage of 0 mV by 1s voltage ramp from -100 to +100 mV with pipette pH (pH_i_) at 7.2. The sperm were bathed initially in HS (for divalent currents) and then DVF (for monovalent *I*_*CatSper*_ currents) before subsequent addition of CCR6 ligands, rDEFB1 (1-500 ng/mL, A-E) or CCL20 (50 ng/ml, F-G) into the DVF bath. CatSper inhibitor mibefradil (40 μM, B) or CCR6 neutralizing antibody (CCR6-Ab, 20 μg/ml) were added prior to addition of CCR6 ligands. The currents elicited at -80 and +80 mV were used for statistical comparisons between different conditions (C, E & G). Data are means ± SEM normalized to the current value in DVF. n (number of measured sperm) = 7 (B), 6 (C) and 5 (E & G) (* p<0.05, ** p<0.01, *** p<0.001, One-way ANOVA). **(H-J)** Patch-clamp recording (H) and statistical summary (I & J) of whole-cell currents elicited by voltage ramp (-100 to +100 mV from holding of 0 mV, 1s) in HS or DVF with subsequent addition of CCL20 (50 ng/ml) or rDEFB1 (500 ng/ml) in sperm obtained from an infertile patient with mutation in CatSper2 gene (I) or from normal individuals (J). Data are means ± SEM. n≥4 (I) and 6 (J) (* p<0.05, One-way ANOVA)

To examine the direct effect of DEFB1 and CCL20 on CatSper channel activity, we assessed their effect on CatSper current (*I*_CatSper_) by whole-cell patch clamp technique with access resistance ranged from 10-15 MΩ. A characteristic of CatSper channel is the absence of *I*_CatSper_ when recording in the 2 mM Ca^2+^-containing extracellular normal physiological solution (HS) even under alkaline intracellular environment (pH 7.2) [4]. However, when perfusing with the divalent-free solution (DVF), where Ca^2+^ was chelated by 5 mM EGTA, a sizeable monovalent *I*_CatSper_ can be recorded [12]. Thus, we investigated the effect of DEFB1 on CatSper under DVF condition. As shown in Figure 2A, rDEFB1 exerted a stimulating effect on CatSper at a concentration as low as 1 ng/ml and showed a half activation around 20 ng/ml. The maximum concentration of rDEFB1 tested in this set of experiments (500 ng/ml) induced a 1.66±0.35 fold increase in *I*_CatSper_ at +100 mV. To measure rDEFB1-induced *I*_CatSper_ at physiological range of potentials, we analyzed the current amplitude at -80 mV and +80 mV respectively. Consistent with previous results, rDEFB1 induced sizable *I*_CatSper_ which could be blocked by mibefradil (30 μM) (Figure 2B-2C) or CCR6 antibody (Figure 2D-2E) at -80 mV. On the contrary, rDEFB1 at 100 ng/ml, which increased CatSper current substantially (Figure 2A), had no effect on potassium channel KSper (Supplementary Figure 1), suggesting that DEFB1 specifically activates CatSper. Similar to the results obtained from DEFB1 treatment, CCL20 also evoked *I*_CatSper_, which was blunted by CCR6 antibody treatment (Figure 2F-2G), suggesting that binding of CCL20 to its receptor CCR6 also activates CatSper channel.

To confirm that the currents induced by DEFB1 and CCL20 were genuinely mediated by CatSper channel, we performed patch-clamp experiments on sperm samples donated by an infertile male who carries a G to C mutation of CatSper2 in one allele and complete absence of CatSper2 in the other allele. The patient’s spermatozoa were demonstrated to have no detectable CatSper current, as evidenced by a constant current amplitude upon switching extracellular solution from 2 mM Ca^2+^-containing HS to DVF (Figure 2H-2I). As expected, treating these CatSper-lacking sperm with either rDEFB1 or CCL20 in DVF solution did not induce detectable current (Figure 2I) while the same treatment in normal sperm induced sizeable *I*_CatSper_ (Figure 2J). Together, these results indicate that the two CCR6 ligands indeed activate CatSper current.

### CCR6 and CatSper form a receptor complex for different ligands

**Figure 3:**
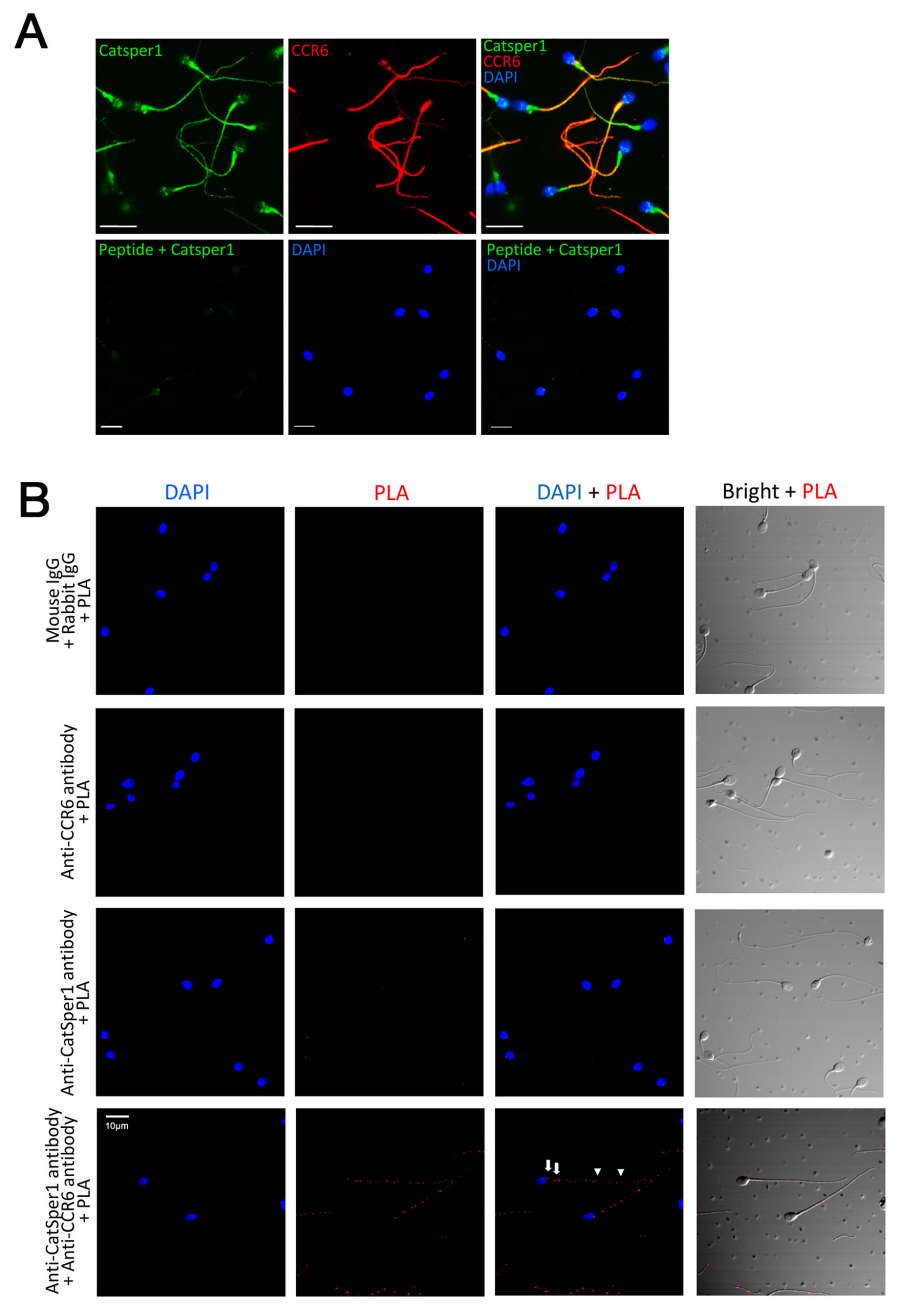
CCR6 interacts with CatSper1 in human sperm **(A)** Representative fluorescence images showing the localization of CatSper1 (green) and CCR6 (red) in the principal and mid-piece of normal human sperm. *Bottom panel,* the antibody against CatSper1 was pre-absorbed with an antigen peptide before staining. Nuclei were counterstained with DAPI (blue). Bars = 10 μm. **(B)** Representative proximity ligation assay (PLA) images showing the close proximities of CatSper1 and CCR6 (bottom right) in the principal (arrow heads) and mid-piece (arrows) of normal human sperm. Sperm samples labelled with normal IgGs with PLA (top left), anti-CCR6 antibody with PLA (top right) or anti-CatSper1 antibody with PLA (bottom left) were used as control. Bars = 10 μm.

The observation that the DEFB1/CCL20-induced Ca^2+^ influx or CatSper current could be blocked by either CCR6 antibody or CatSper inhibitors indicates that both CCR6 and CatSper are required for mediating the DEFB1/CCL20 action. This prompted us to speculate that CCR6 and CatSper might interact or at least locate in close proximity to regulate Ca^2+^ influx. To test this, we employed immunofluorescence staining to examine the localization CCR6 and CatSper1, a major pore-forming subunit of the CatSper channel [2, 3]. Consistent with previous report [5], CCR6 was localized to the mid-piece and principal piece in human sperm (Figure 3A). In addition to the previously reported localization in the principal piece [2], CatSper1 was also localized to the mid-piece and co-localized with CCR6 (Figure 3A). CatSper1 immunofluorescent signal was absent in sperm stained with blocking peptide-pretreated antibody, indicating the staining was specific (Figure 3A). We further used proximity ligation assay (PLA) to examine the potential interaction between CCR6 and CatSper1 [23, 24]. Consistent with the immunofluorescent staining results, PLA signals, which indicates the close proximity of CCR6 and CatSper1, were observed at the mid-piece and principal piece (Figure 3B).

**Figure 4:**
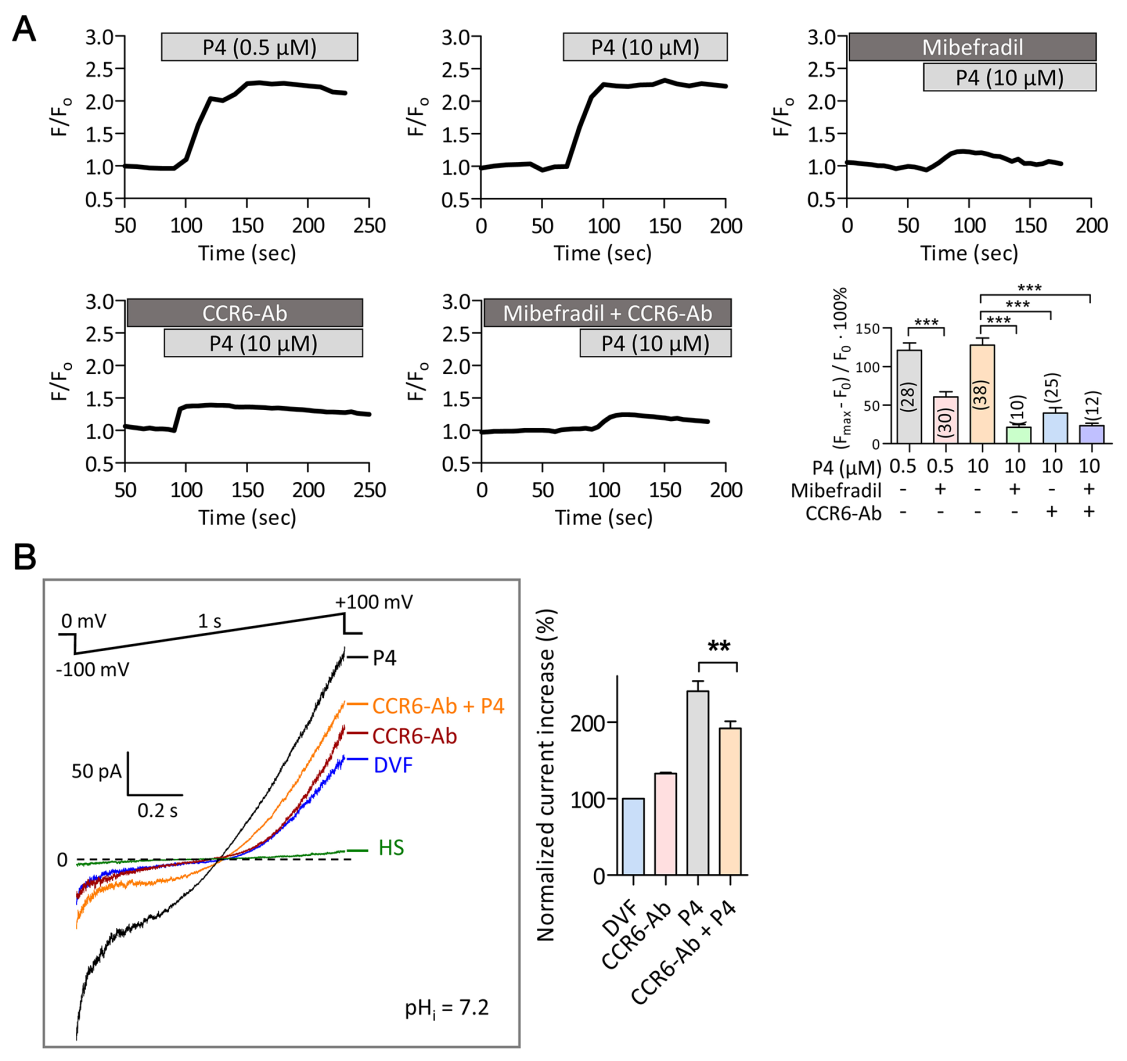
Involvement of CCR6 receptor in progesterone-induced Ca^2+^ influx in human sperm **(A)** Time-course change in Fluo-4 (F) normalized to the initial intensity (F_0_) in normal human sperm in response to progesterone (P4, 0.5-10 μM) in the presence or absence of mibefradil (40 μM), CCR6-Ab (20 μg/ml) or both. *Bottom right,* statistic summary of P4-induced Ca^2+^ responses as indicated by the difference (in % F_0_) between F_0_ and the maximal florescence intensity (F_max_) after P4 addition. Data are presented as mean ± SEM. Number of measured sperm is indicated in each column (***p<0.001, One-way ANOVA). **(B)** Patch-clamp recording of whole-cell currents elicited by voltage ramp (-100 to +100 mV from holding of 0 mV, 1s) bathed in HS or DVF in the absence or presence of a CCR6 neutralizing antibody (CCR6-Ab, 20 μg/ml) with subsequent addition of P4 (500 nM). Pipette pH (pHi) =7.2. Statistic summary is shown on the right panel. Data are presented as mean ± SEM. n≥7 (** p<0.01, One-way ANOVA).

The close proximity of CCR6 receptor and CatSper channel and their involvement in mediating Ca^2+^ influx induced by physiological ligands DEFB1 and CCL20 prompted us to further investigate their possible involvement in mediating the response elicited by one of the best-known physiological factors in the female tract, progesterone, which has been previously shown to activate the CatSper channel and lead to Ca^2+^ influx [12, 13]. The Ca^2+^ imaging results showed that addition of 500 nM or 10 μM progesterone triggered an acute increase in [Ca^2+^]_i_, which could be inhibited by CatSper inhibitor, mibefradil (40 μM) (Figure 4A). Of note, the magnitude of [Ca^2+^]_i_ elevation induced by progesterone was significantly higher than that induced by DEFB1 or CCL20 (Figure 1). CCR6 neutralizing antibody (20 μg/ml) also inhibited the progesterone-induced Ca^2+^ elevation (Figure 4A). Blocking the CCR6 receptor and inhibiting CatSper simultaneously did not produce significant additive effect in inhibiting the [Ca^2+^]_i_ elevation (Figure 4A), indicating that both CCR6 and CatSper are likely to be involved in the same pathway. In the patch-clamp experiments, CCR6 antibody also inhibited progesterone-induced *I*_*CatSper*_ but not basal CatSper current (Figure 4B), excluding possible non-specific effect of the CCR6 antibody on CatSper channel. Thus, these results suggest that the CCR6 is also required for the progesterone-induced CatSper activation.

### CCR6 and CatSper act-in-concert in maintaining sperm functions

**Figure 5:**
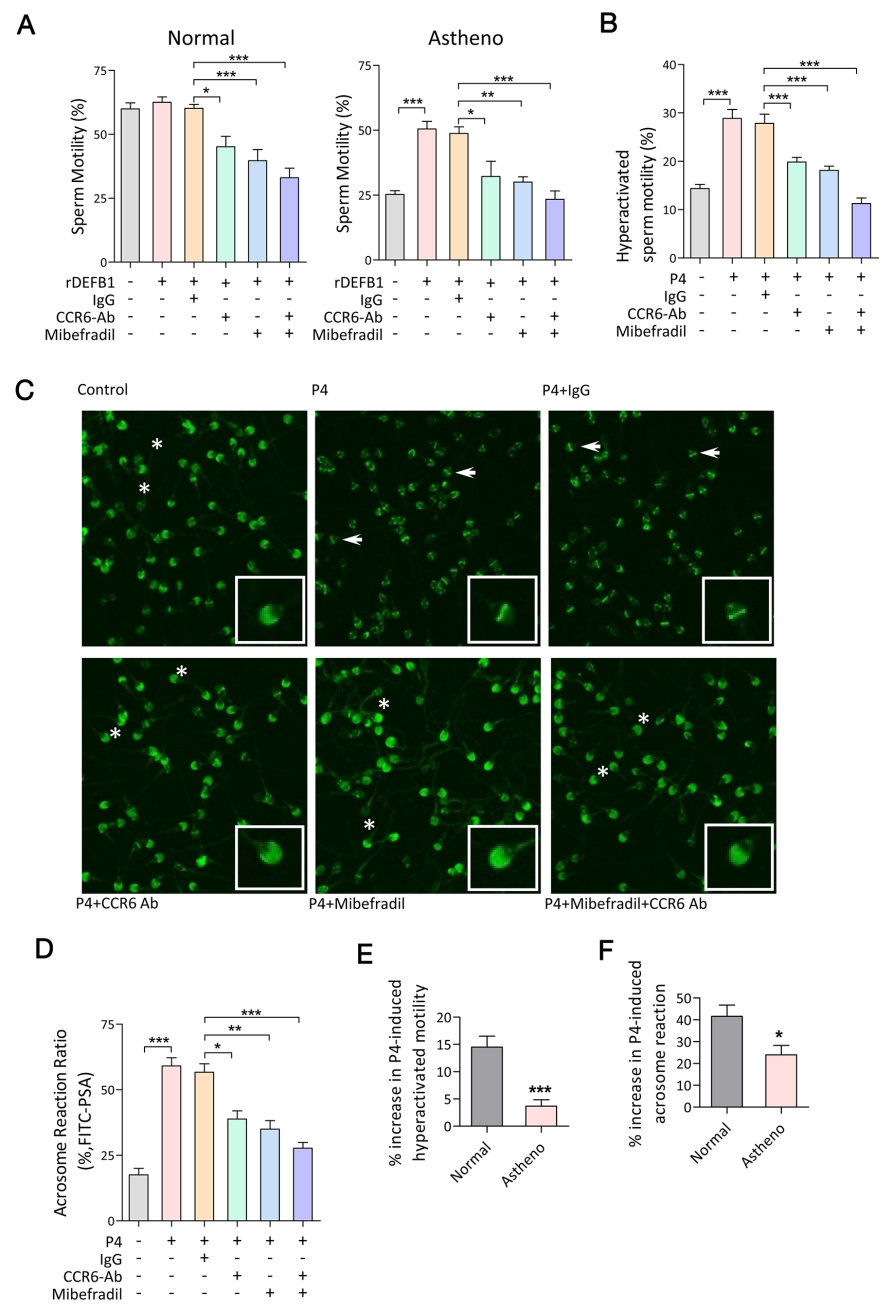
CCR6 and CatSper act in concert in mediating Ca^2+^-influx-dependent sperm functions **(A & B**) Computer-assisted sperm analysis (CASA) measurement of DEFB1-induced motility (A, n≥11) and progesterone-induced hyperactivated motility (B, n≥16) in sperm samples obtained from normal or asthenozoospermia patients in the presence or absence of the CCR6 neutralizing antibody (CCR6-Ab, 20 μg/ml) and/or CatSper inhibitor (mibefradil, 40 μM). **(C & D)** Representative fluorescence images of PSA staining (C) and quantification of acrosome reaction (D, n=10) in indicated sperm samples treated with progesterone (P4, 10 μM) with or without CCR6-Ab (20 μg/ml) and/or mibefradil (40 μM). Acrosome-intact and acrosome-reacted sperm were marked by asterisks and arrows respectively. Enlarged images are shown in insets. **(E & F)** Quantification of percentage increase in hyperactivated motility (E, n≥11) and acrosome reaction (F, n=10) induced by P4 (10 μM) in sperm obtained from normal and asthenozoospermia patients. Data are presented as mean ± SEM. * p<0.05, ** p <0.01, *** p<0.001. One-way ANOVA (A, B and D), Student *t* test (E and F).

Finally, we examined the role of CCR6 and CatSper in sperm functions. In the first set of experiments, we examined the effects of CCR6 and CatSper on DEFB1-induced sperm motility. Normally, during sperm transit through the epididymis, DEFB1, produced and released by the epididymal epithelial cells, binds to sperm and induces motility [5]. Adding rDEFB1 to normal human sperm could not induce significant increase in sperm motility, however, CCR6 antibody (20 μg/ml) or CatSper inhibitor mibefradil (40 μM) could each significantly decreased the forward motility, with no significant additive effect observed when used in combination (Figure 5A), suggesting that both CCR6 and CatSper are required for sustaining normal sperm motility. We further examined DEFB1-induced motility in infertile spermatozoa from asthenozoospermia patients, who had lowered level of DEFB1 (Supplementary Figure 2), as reported previously [5]. Treatment of the patients’ spermatozoa with rDEFB1 (800 ng/ml) restored the motility to above 50% (Figure 5A), and the rDEFB-rescued motility could be blunted by either CCR6 antibody or CatSper inhibitor with no significant additive effect observed when used in combination (Figure 5A). Similar results were obtained in normal and infertile sperm treated with CCL20 (Supplementary Figure 3). Together, these results suggested that CCR6 and CatSper act-in-concert in mediating DEFB1 and CCL20-induced Ca^2+^ influx required for sperm motility.

In human sperm, progesterone induces hyperactivation and acrosome reaction [14]. If the CCR6 and CatSper are both involved in mediating the effect of progesterone in these processes, we would expect the progesterone effect to be blunted by either CCR6 antibody or CatSper inhibitor. Indeed, the effects of progesterone on hyperactivated motility (Figure 5B) and the acrosome reaction (Figure 5C-5D) were blocked by either the CCR6 neutralizing antibody and/or CatSper inhibitor (Figure 5B-5D). Taken together, these results suggest that similar to the DEFB1-induced sperm motility, both CCR6 and CatSper are also required for progesterone-induced hyperactivation and the acrosome reaction. This led us to speculate that spermatozoa obtained from asthenozoospermia patients with reduced levels of either CCR6 [5] or CatSper [25] (Supplementary Figure 2) should also exhibit defects in hyperactivation and acrosome reaction. To test this, we examined progesterone-induced hyperactivation and acrosome reaction in spermatozoa from the same cohort of asthenozoospermia patients, and the results showed that both the progesterone-induced hyperactivation and acrosome reaction were significantly impaired in patients’ spermatozoa compared to those obtained from normal men (Figure 5E-5F), confirming an important role of the CCR6 and CatSper in sperm functions.

## DISCUSSION

The present study has identified a previously unexpected role of CCR6 receptor and CatSper channel in human sperm that act-in-concert in mediating ligand-induced Ca^2+^ responses required for various sperm functions. This finding provides a mechanism explaining the recently discovered role of DEFB1 in regulating sperm motility [5]. Our previous study has demonstrated that CCR6 is a receptor for DEFB1 in human sperm and that the binding of DEFB1 to CCR6 can result in cytosolic Ca^2+^ elevation and increase in motility. However, it was not clear how DEFB1 could trigger Ca^2+^ influx. The present study has provided strong evidence for the involvement of CatSper in a complex with CCR6 that mediates the CCR6 ligands (DEFB1 and CCL20)-induced Ca^2+^ influx or *I*_*CatSper*_, as well as DEFB1/CCL20-induced sperm motility. More surprisingly, the presently identified a role of CCR6 in mediating, at least in part, the effect of progesterone on Ca^2+^ influx or *I*_*CatSper*_, as well as progesterone-induced hyperactivation and acrosome reaction. The receptor for mediating the non-genomic effects of progesterone has long been under intense debate and recent studies have demonstrated that progesterone interacts with orphan enzyme ABHD2, which act as a progesterone receptor in sperm. [16, 17]. Our findings suggest either that CCR6 acts as a co-factor in the progesterone-ABHD2 complex or that CCR6 mediates the progesterone-induced CatSper activation through an alternative pathway. Further study is required to test these possibilities. Nonetheless, we show that CCR6 is required for CatSper1-dependent Ca^2+^ influx induced by different ligands, including progesterone, in addition to DEFB1 and CCL20, and perhaps more. This in-concert role of CCR6 and CatSper appears to be multifunctional and responsible for the various ligand-induced Ca^2+^ responses required for sperm motility, hyperactivation and the acrosome reaction.

It has been shown that the motility of epididymal sperm increase progressively *in vitro* from the caput (head) towards the cauda (tail) region. However, *in vivo*, the sperm become quiescence for storage in the cauda possibly through a controlling/repressing mechanism [26]. Since DEFB1 is also expressed in the epididymis, if the binding of DEFB1 to CCR6 receptor activates the CatSper channel required for motility, this pathway may be subjected to similar controlling mechanism to facilitate the storage of sperm. In this scenario, the CCR6 - CatSper complex may be the target for the control of sperm motility during the storage period. After the deposit of sperm into the female reproductive tract, the absence of controlling mechanism allows the sperm to regain motility upon the binding of female tract-derived DEFB1 and other CCR6 ligand that activate the CatSper channel.

CCR6 is a G-protein coupled receptor that triggers GTP-dependent intracellular signaling upon binding to its ligand [27]. Interestingly, the *I*_*CatSper*_ induced by binding of DEFB1/CCL20 and possibly progesterone to CCR6 receptors do not require additional GTP in the pipette solution, suggesting either that the endogenous GTP is sufficient for the activation of CatSper channel or that CCR6 activates CatSper through a GTP-independent mechanisms [28]. Since CCR6 and CatSper co-localized to the same region on sperm, we propose that the binding of ligands to CCR6 receptor triggers conformational change that directly activates CatSper channel. This notion is supported by the results that blocking CCR6 receptors inhibits the CatSper-dependent Ca^2+^ influx induced by progesterone where intracellular signaling is very unlikely to be involved due to the rapid induction time [12, 13].

**Figure 6:**
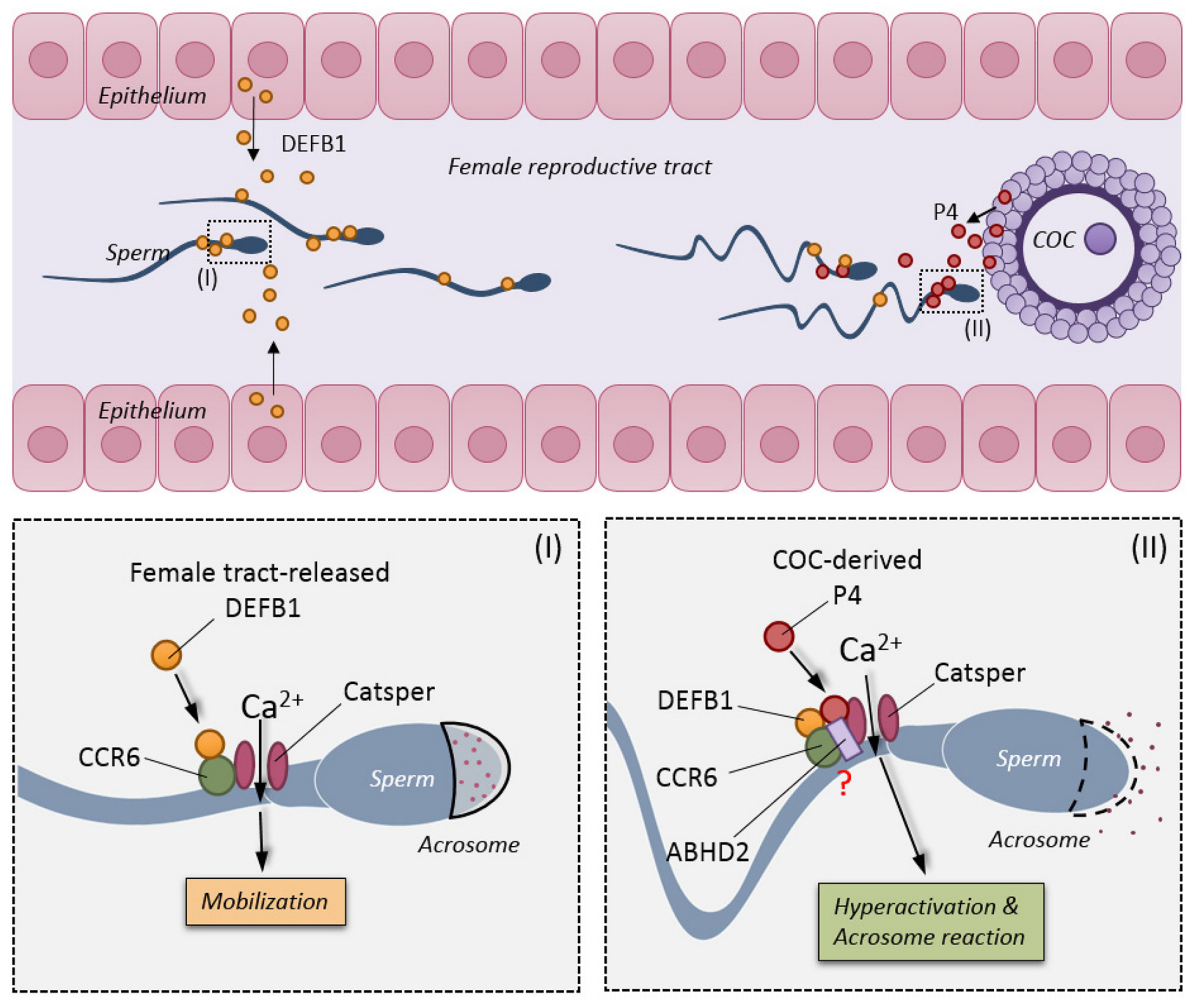
Schematic diagram showing the physiological roles of CCR6 receptor and CatSper channel in sperm In the female reproductive tract, DEFB1 secreted from the uterine epithelium binds to CCR6 receptor and elicits a CatSper-dependent Ca^2+^ influx required for maintaining motility (bottom left). When sperm approach the fertilization site, progesterone secreted from the cumulus-oocyte-complex binds to the CCR6/CatSper complex and triggers an acute and stronger CatSper-dependent Ca^2+^ influx that is essential for hyperactivation and acrosome reaction (bottom right).

In the female reproductive tract, it has been reported that DEFB1 is secreted by the uterine epithelium and progesterone from the cumulus-oocyte complex [14, 15, 29]. The presently demonstrated ability of these spatially differentially expressed/released physiological factors to activate CCR6 receptor and CatSper channel in human sperm suggests a sequential induction mechanism for a series of events leading to fertilization (Figure 6). For example, DEFB1, which is expressed in the female reproductive tract [30], sustains motility and helps sperm travel through the uterus, and then, when sperm are approaching the oviduct, progesterone released from the egg complex induces hyperactivation and the acrosome reaction that allow the sperm to fertilize the egg. Of note, progesterone induces a much more rapid and stronger Ca^2+^ response compared to that induced by DEFB1 and CCL20. It appears that while the CCR6 and CatSper can be activated by different ligands, the gating of CatSper or the magnitude of the Ca^2+^ response may depend on the type of ligands that sperm encounter. It is plausible that the CCR6 receptor may undergo ligand-dependent conformational changes, which may result in differential CatSper channel gating. Further work is required to test this possibility. The differential Ca^2+^ responses, both in the kinetics and magnitude, induced by different ligands, as currently demonstrated for DEFB1, CCL20 and progesterone, may explain how the CCR6 and CatSper are able to regulate different sperm functions essential for fertilization.

The importance of the CCR6 receptor and CatSper channel can be highlighted by the observed reduction in the expression levels of either CCR6 [5] or CatSper [25] or both in sperm from the infertile patients recruited in the current study, indicating that defects in any component of this receptor-channel module with various sperm malfunctions may contribute to different forms of male infertility. Notably, all sperm samples from the infertile patients cohort recruited in this study express lowered level, but not complete absence of CCR6 and CatSper1. Thus, it is plausible that the rDEFB1 fully activates the remaining receptor-channel module in restoring motility in patient’s sperm. Despite the important roles of CCR6/CatSper receptor-channel module in human sperm functions, CCR6 KO mice have been shown to be fertile [31]. It should be noted that fertilization appears to be quite distinct between mice and human [1]. In fact, the effect of progesterone in activating CatSper channel was only observed in human sperm but not mouse sperm [12]. Further, while we observed an essential role of DEFB1 in regulating the motility of human sperm and defending male fertility in humans [5], DEFB1 KO mice are fertile [32]. Thus, it is likely that the presently observed effects of these physiological ligands on CCR6 and CatSper are specific for human sperm.

In summary, the present study has revealed pivotal role of CCR6 receptor and CatSper channel in human sperm serving as a receptor-channel module for various physiological factors, including defensins and progesterone, that regulate a spectrum of sperm functions required for fertilization. The present study provides the evidence that CatSper channel activity can be modulated by different ligands through its proximal receptor CCR6. The presently demonstrated critical role of the CCR6 and CatSper in mediating ligand-induced Ca^2+^ influxes required for various sperm functions suggests that CCR6 and CatSper could be markers for diagnosis of male infertility and a potential target for fertility control.

## MATERIALS AND METHODS

### Reagents

Rabbit IgG (sc-2027), rabbit anti-human CatSper 1 (H300) (sc-33153), rabbit anti-CatSper 1 (Santa Cruz, sc-33153), rabbit anti-human CCR 6 (H-81) (sc-5623), goat anti-human CCR-6 (N-19) (sc-9695), mouse anti-human CCR6 (R&D MAB195), and rabbit anti-human DEFB1 (FL-68) (sc-20797) antibodies were purchased from Santa Cruz Biotechnology (Santa Cruz, CA). Recombinant human active DEFB1 (ab50048) was purchased from Abcam (Cambridge, MA). CCL20 (360-MP-025/CF) was purchased from R & D Systems. Quinn’s human tubal fluid (HTF) (ART-1020) media was purchased from SAGE Media. Mouse monoclonal anti-β-actin (1:5000, A1978), CatSper1 ion channel activator Progesterone (P8783), CatSper1 ion channel inhibitor Mibefradil (M5441) and NNC 55-0396 hydrate (N0287) were purchased from Sigma-Aldrich Co (St Louis, MO). Discontinuous density gradient Percoll (17-0891-01) was purchased from GE Healthcare Life Sciences (Pittsburg, PA).

### Clinical samples

The use of human specimens for this project was approved by the Regional Committee for Medical Research Ethics and the Human Ethics Committee of Shenzhen Second People’s Hospital, and written consent was obtained from each subject.

Semen was obtained from healthy donors or infertile patients at Shenzhen Second People’s Hospital according to World Health Organization Criteria [33] or at Human Sperm Bank in Jiangxi province and Maternal and Child Care Service Centre in Jiangxi province on the premise of voluntary. Subjects with a family history of endocrine or anatomical disorders were excluded from the study. Semen collection was done by masturbation after 3-5 day-sexual abstinence.

After complete liquefaction at 37°C for 30 minutes, parameters including semen volume, sperm concentration, sperm motility, sperm viability and sperm morphology were analyzed by a computer assisted semen analysis (CASA) system under 200 × magnification. Normal controls were selected according to the following criteria: spontaneous pregnancy achieved within less than one year of pregnancy expectation and with the last pregnancy less than one year prior to study; total sperm motility (Progressive motility + Non-progressive motility, PR+NP)≥40% (38-42%, 5^th^ centile, 95% Cl), and sperm progressive motility (PR)≥32% (31-34%, 5^th^ centile, 95% Cl). Subjects with sperm progressive motility (PR) <32% were regarded as asthenozoospermia.

For the CatSper-lacking sperm sample, the number of sperm was 35.02 ×10^6^ cells/ml, of which 80.21% were motile. The percentage of PR (sperm with progressive and rapid motility) and NP (sperm with non-progressive motility) were respectively 52.78% and 19.79%. Average path velocity (VAP) was 15.32 μm/s. Curvilinear velocity (VCL) was 10.76 μm/s. Straight line velocity (VSL) was 20.47 μm/s. Amplitude of lateral head displacement (ALH) was 0.55 μm. And linearity of progression (LIN) was 52.55%.

### Sperm motility assay by CASA

After Percoll density gradient centrifugation, human sperm samples from normal and infertile patients were washed and adjusted to 1 x 10^6^ cells/ml using the Neubauer cell chamber in HTF (3% BSA). The samples were incubated with either rDEFB1(800 ng/ml), CCL20 (50 ng/ml) or progesterone (10 μM) for one hour at 37°C under 5% CO_2_ with or without anti-CCR 6 antibody (20 μg/ml) and/or CatSper1 inhibitor (Mibefradil, 40 μM) for further 15min at 37°C. 15% glycerol in PBS and normal rabbit IgG were included as vehicle controls.

For CASA analysis, 4 μl of sperm suspension at a concentration of 1X10^6^ cells/mL was placed in a Counting Chamber (0.01 mm_2_, 10 μm deep) and assessed for motility characteristics at 37°C. For each sample, 10 randomly-selected fields containing more than 400 motile tracks were examined at 60 Hz. Average values for sperm motion parameters including curvilinear velocity (VCL), straight-line velocity (VSL), average path velocity (VAP), amplitude of lateral head displacement (ALH), linearity (LIN=VSL/VCL), and straightness (STR=VSL/VAP) were recorded. Rapid or slow motile sperm cells were classified according to the CASA standard cutoff values for VAP and VSL. Slow cells were defined as cells with either of the following attributes: VAP < 20 μm/s or VSL < 30 μm/s. Spermatozoa were designated as hyperactive if they had a VCL ≥150 μm/s, ALH ≥7.0 μm/s and LIN ≤50% [34]. The parameters of sperm movement assessed were graded a (the average velocity R25 mm/s) and b (the average velocity between 15 and 25 mm/s). Forward motility was defined by percentage of sperm showing grade a+b motility pattern.

### Immunofluorescence staining and confocal microscopy

Sperm slides were incubated with both rabbit anti-CatSper1 Ab (1:100 dilution, sc-33153) and goat anti-CCR6 Ab (1:100 dilution, sc-9695) overnight at 4°C. After washed three times, slides were incubated with Alexa 594-conjugated donkey-anti-rabbit and Alexa 488-conjugated donkey-anti-goat secondary antibodies for 1h at room temperature. Slides were counterstained with Hoechst 33258 (1 μg/ml, Invitrogen, Camarillo CA) and mounted with Prolong® Gold Antifade Reagent (Invitrogen, Camarillo CA, USA). Primary antibodies pre-incubated with neutralizing peptides were used as negative controls. The slides were then stored in a dark box before visualization. At least 200 sperm were captured randomly under a confocal microscope (Zeiss, Germany) for statistical analysis using Image-Pro Plus 5.1 software.

### Proximity ligation assay (PLA)

Washed human spermatozoa were fixed in 4% formaldehyde for 15 min at 4°C and smeared onto slides. Proximity ligation assays were performed using the Duolink in situ PLA kit (Sigma). Slides were washed in PBS, and permeabilized with 0.1% Triton X-100 in PBS for 5 min. The slides were incubated in blocking solution for 1 hr at room temperature, followed by an overnight incubation with anti-CCR6 (R&D MAB195) (1:50) antibody in conjunction with anti-CatSper (Santa Cruz, sc-33153) (1:25) antibodies. After two washes with PBS, slides were incubated with two oligonucleotide-linked secondary antibodies (PLA probes) for 2 hr at 37 °C. Subsequent hybridization and ligation were carried out for 1 hr and the amplification was carried out at 37 °C for 2 hr. Finally, slides were mounted with Duolin in Situ Mounting Medium with DAPI for 15 mins. Signals were captured using Olympus Fluoview FV1200 confocal microscope.

### Induced acrosome reaction assay (PSA-FITC)

Human sperm samples from normal and infertile patients were adjusted to 1x10^6^ cells/mL using the Neubauer cell chamber in HTF (3% BSA), then incubated for 3 hours at 37°C under 5% CO_2_ in air to induce capacitation. Progesterone (10 μM) was added with or without anti-CCR 6 antibody (20 μg/ml) and/or CatSper inhibitor (Mibefradil, 40 μM)) for further 15min at 37°C. After fixed in ethanol, spermatozoa smear was dried in air and stained using PSA-FITC. About 400 spermatozoa were captured under X 400 magnification with oil immersion at 450-490 nm excitation.

### Intracellular Ca^2+^ measurement

Spermatozoa were incubated with either rDEFB1 (800 ng/ml), CCL20 (50 ng/ml) or progesterone (10 μM), respectively, with or without anti-CCR 6 antibody (20 μg/ml) and/or CatSper1 inhibitor (Miberfiral, 40 μM) at 37°C for 1 hr. Sperm were loaded with 5 uM fluo-4, 1.5 uM Pluornic F-127 in modified sperm washing medium (97.8 mM NaCl, 4.69 mM KCl, 0.2 mM MgSO_4_, 0.37 mM KH_2_PO_4_, 2.04 mM CaCl_2_, 4 mM NaHCO_3_, 21 mM HEPES, 2.78 mM Glucose, 0.33 mM Na pyruvate, 21.4 mM Na lactate and 5mg/ml BSA) in the dark at 32°C for 30 min. After loading, sperm were washed with fresh modified sperm washing medium. 15% glycerol in PBS and normal rabbit IgG were included as vehicle controls.

To start the experiment, sperm suspensions were deposited on the coverslip precoated with poly-L-lysine (0.01% w/v) for 2 minutes. Unattached sperm were removed by gently washing and the chamber was filled with modified washing medium. Measurement was made on an epifluorescence microscope (Nikon Eclipse Ti, Japan) with a 60 X oil objective lens (1.40 NA) (Nikon, Japan) and a CCD camera (Spot Xplorer, USA) controlled by the software MetaFluor (Universal Imaging). Light was provided by the Xenon lamp (Hamamatsu, Japan). Excitation at 488 nm was used and emission was collected at 510 nm. Changes in intracellular calcium level were calculated by percentage change of peak fluorescent intensity after treatment (F_max_) – basal fluorescent intensity (F_0_)/F_0_.

### Whole-cell Patch-clamping recording

After liquefaction for 1 hr, 0.5 ml standard bath solution (310 mOsm-HS) containing (in mM): 135 NaCl, 5 KCl, 1 MgSO_4_, 2 CaCl_2_, 5 glucose, 1 Na pyruvate, 10 lactic acid, 20 HEPES (pH 7.4) was added to 1 ml semen. The resulting suspension was centrifuged at 500 g for 6 min. Pellet was washed with fresh 1.5 ml HS solution and centrifuged again. Spermatozoa were then resuspended in 1 ml HS and used for electrophysiological recordings.

All gigaohm seals (>10 GΩ) between the patch pipette and human uncapacitated spermatozoa were formed at the cytoplasmic droplet in standard bath solution (HS) containing (in mM): 135 NaCl, 5 KCl, 1 MgSO_4_, 2 CaCl_2_, 5 glucose, 1 Na pyruvate, 10 lactic acid, 20 HEPES adjust to pH 7.4 with NaOH, 310 mOsm/L. HS solution was also used to record baseline current while measuring monovalent CatSper currents (Ca2+ in HS solution inhibits monovalent CatSper currents and causes Ca2+-dependent inactivation of CatSper channels). Monovalent currents were recorded by perfusing with a sodium-based divalent-free solution (DVF) containing (in mM): 150 NaCl, 20 HEPES, and 5 EGTA adjusted to pH 7.4 with NaOH, perfusion time usually was more than 45 s. Pipettes (15−20 MΩ) for whole-cell patch-clamp recordings of CatSper currents were filled with 135 Cs-Mes, 10 HEPES, 10 EGTA, 5 CsCl adjusted to pH 7.2 with CsOH. Some KSper recordings were performed with pipette solutions containing (in mM): 155 KOH, 5 KCl, 20 Hepes, 115 Mes, 10 BAPTA adjusted pH to 8.0 with KOH. And the bath solution was 160 mM KOH, 10 mM Hepes, 150 mM Mes, and 2 mM Ca(Mes)_2_, adjusted to pH 7.4 with Mes. Solutions were applied directly via a local perfusion system allowing switching between different test solutions. Solution exchange time with this system is typically <0.5 s. Current waveforms were analyzed either with Clampfit or Grapher 8. All experiments were at room temperature (∼22–25 °C).

### Statistical analysis

Statistical analysis was calculated using GraphPad Prism version 5.0 (GraphPad Software, San Diego, CA) or SPSS version 17.0. Results are given as mean ± s.e.m. Comparisons were subjected to t-test (for two-group comparisons), one-way analysis of variance (for multi-group comparisons), or two-way analysis of variance with Bonferroni post hoc tests. Statistical significance was set at p < 0.05.

## SUPPLEMENTARY MATERIALS FIGURES


