# Supplementary material for: CCR6 is required for ligand-induced CatSper activation in human sperm
Source: Oncotarget. 2017 Sep 5;8(53):91445–58. doi: 10.18632/oncotarget.20651 (PMC5710936; doi:10.18632/oncotarget.20651)
Supplement: Supplementary file 1 [file oncotarget-08-91445-s001.pdf]

CCR6 is required for ligand-induced CatSper activation in human sperm

SUPPLEMENTARY MATERIALS

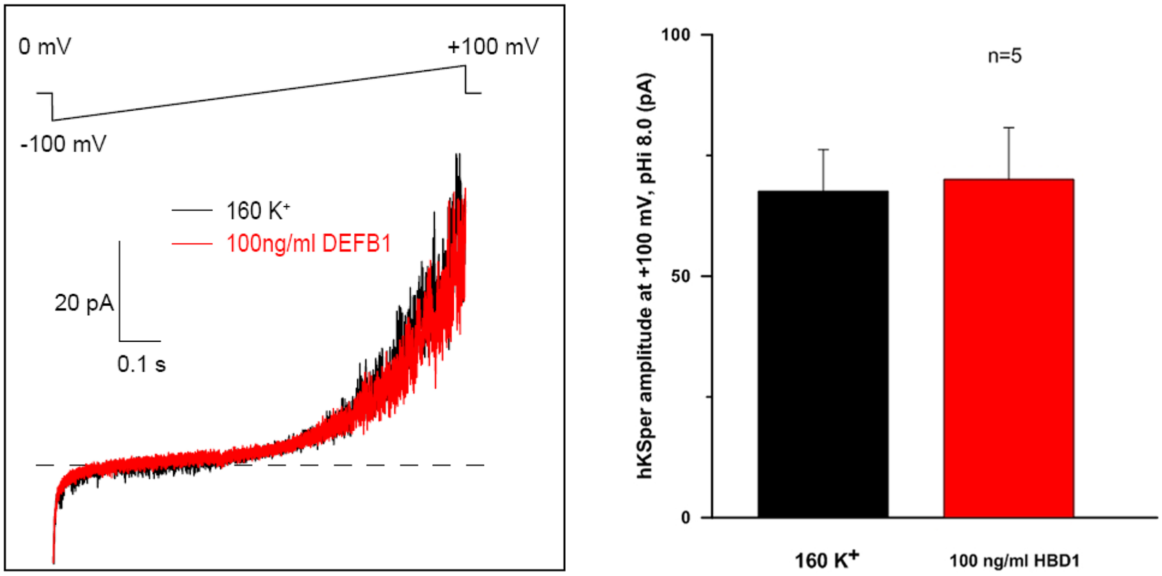

Supplementary Figure 1: DEFB1 did not activate sperm potassium channel. Voltage-current relation in sperm treated with 100 ng/ml rDEFB1 (left panel). Quantification of potassium current with or without rDEFB1 treatment (right panel, n=5).

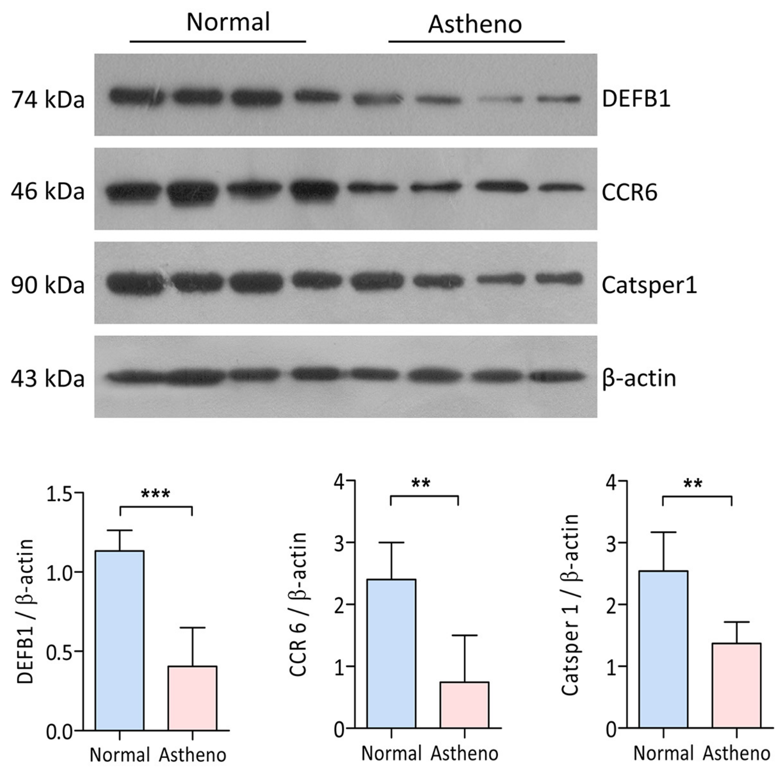

Supplementary Figure 2: Protein level of DEFB1, CCR6 and CatSper1 is lowered in sperm obtained from asthenozoospermia patients. Representative Western blot of CCR6, DEFB1 and CatSper1 in sperm obtained from normal individuals and asthenozoospermia patient (n=5 in each group) with quantification of each group shown in bottom panel. β-actin was used as loading control. Data are presented as mean ± SEM. Student's *t* test * $p<0.05$, ** $p<0.01$, *** $p<0.001$.

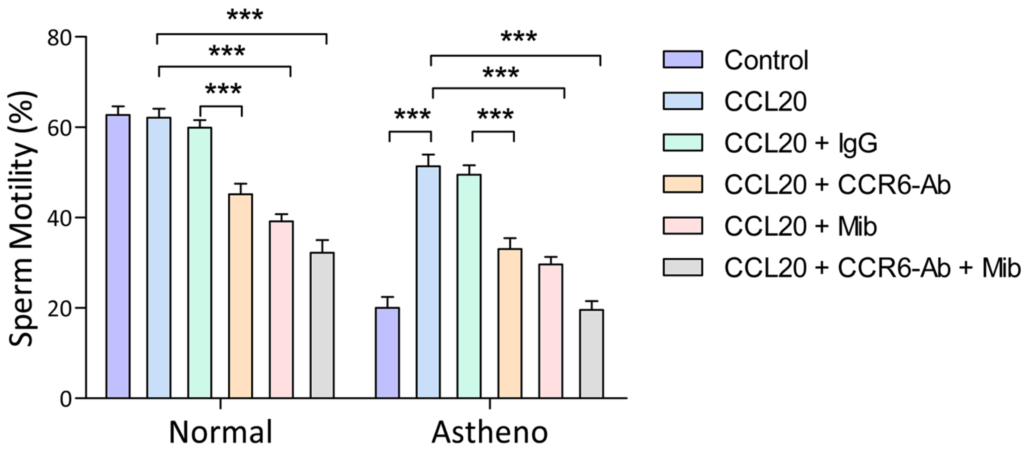

Supplementary Figure 3: CCR6 receptor and CatSper channel are both involved mediating CCL20-induced sperm motility. CASA measurement of forward motility in sperm samples obtained from normal and infertile patients treated with CCL20 (50 ng/ml) in the presence or absence of CCR6 neutralizing antibody (20 µg/ml) and/or CatSper inhibitors (mibefradil – 40 µM) ($n\geq12$). Data are presented as mean ± SEM. One-way ANOVA * $p<0.05$, ** $p<0.01$, *** $p<0.001$.
